# Lyophilized cell-free supernatants of *Limosilactobacillus fermentum* T0701 exhibited antibacterial activity against *Helicobacter pylori*

**DOI:** 10.1038/s41598-024-64443-4

**Published:** 2024-06-13

**Authors:** Phoomjai Sornsenee, Komwit Surachat, Thanawin Wong, Apichat Kaewdech, Morteza Saki, Chonticha Romyasamit

**Affiliations:** 1 Department of Family and Preventive Medicine, Faculty of Medicine, Prince of Songkla University, Songkhla, 90110 Thailand; 2 Department of Biomedical Sciences and Biomedical Engineering, Faculty of Medicine, Prince of Songkla University, Hat Yai, Songkhla, 90110 Thailand; 3 Gastroenterology and Hepatology Unit, Division of Internal Medicine, Faculty of Medicine, Prince of Songkla University, Songkhla, Thailand; 4 Department of Microbiology, Faculty of Medicine, Ahvaz Jundishapur University of Medical Sciences, Ahvaz, Iran; 5 Department of Medical Technology, School of Allied Health Sciences, Walailak University, Nakhon Si Thammarat, 80160 Thailand; 6 Center of Excellence in Innovation of Essential Oil and Bioactive Compounds, Walailak University, Nakhon Si Thammarat, 80160 Thailand; 7 Research Center in Tropical Pathobiology, Walailak University, Thasala District, Nakhon Si Thammarat, Thailand

**Keywords:** *Helicobacter pylori*, *Limosilactobacillus fermentum T0701*, Antibiotic resistance, Antibiotic, Cell-free supernatant, Cytotoxicity, Gastrointestinal diseases, Infectious diseases, Molecular medicine, Microbiology, Antimicrobials

## Abstract

*Helicobacter pylori* is a prominent gastrointestinal pathogen associated with various gastrointestinal illnesses. It presents substantial health risks due to its antibiotic resistance. Therefore, it is crucial to identify alternative treatments for *H. pylori* infections. *Limosilactobacillus* spp exhibit probiotic properties with beneficial effects in humans; however, the mechanisms by which it counteracts *H. pylori* infection are unknown. This study aimed to evaluate the potential of *Limosilactobacillus fermentum* T0701 lyophilized cell-free supernatants (LCFS) against *H. pylori.* The LCFS has varying antimicrobial activities, with inhibition zones of up to 10.67 mm. The minimum inhibitory concentration and minimum bacterial concentration of LCFS are 6.25–25.00 mg/mL and 6.25 mg/mL to > 50.00 mg/mL, respectively, indicating its capability to inhibit *H. pylori.* There is morphological damage observed in *H*. *pylori* treated with LCFS. Additionally, *H. pylori* adhesion to AGS cells (human gastric adenocarcinoma epithelial cells) reduces by 74.23%, highlighting the LCFS role in preventing bacterial colonization. Moreover, LCFS exhibits no cytotoxicity or morphological changes in AGS cells, and with no detected virulence or antimicrobial resistance genes, further supporting its safety profile. *L. fermentum* T0701 LCFS shows promise as a safe and effective non-toxic agent against *H. pylori,* with the potential to prevent gastric colonization.

## Introduction

*Helicobacter pylori* is a gram-negative, microaerophilic, motile, spiral-shaped bacterium that predominantly colonizes the human gastric mucosa and plays a pivotal role in a wide spectrum of gastrointestinal pathologies, including asymptomatic to chronic gastritis, peptic ulcers, duodenal ulcers, mucosa-assisted lymphoid tissue lymphoma, and gastric cancer^1,2^. *H. pylori* infection is prevalent globally, affecting almost 50% of the world's population, with transmission often associated with the fecal–oral pathways^2^. From 2015 to 2022, the global prevalence of *H pylori* infection was 43.7% in adults and 34.4% in children and adolescents^3^. Moreover, in 1994, *H. pylori* was categorized as a Group 1 carcinogen by the World Health Organization International Agency for Research on Cancer^4^.

The pathogenesis of *H. pylori* involves a sophisticated mechanism that allows it to survive and thrive in the highly acidic environment of the stomach, such as flagella, urease, and toxins. Additionally, two major virulent factors of *H. pylori* have been reported to induce pathogenesis: cytotoxin-associated gene A (*CagA*), which is delivered to host cells via the type IV secretion system, and vacuolating cytotoxin A (*VacA*). These virulence factors disrupt cell signaling and induce inflammation, leading to tissue damage and ulcer formation. Chronic inflammation triggered by persistent infection can contribute to the development of atrophic gastritis and increase the risk of gastric cancer^1,2,5^. Currently, the main strategy for eradicating *H. pylori* involves a combination of oral antibiotics, proton pump inhibitors (PPIs), and bismuth agents. However, this treatment is not always effective; in 10–20% of cases, *H. pylori* is not eradicated after treatment, leading to recurrent *H. pylori* infection. Moreover, the antibiotic resistance rate of *H. pylori* has increased and the adverse effects of eradication therapy can be severe. Therefore, there is an urgent need to develop novel, inexpensive, alternative, and implementable strategies to combat *H. pylori* infection, such as new anti-*H. pylori* drugs.

Probiotics are live microorganisms with low or no pathogenicity, which confer health benefits to the host when administered in adequate amounts^6^. Currently, the clinical benefits of probiotics are widely accepted, and their therapeutic applications include disorders, such as diarrhea, antibiotic-associated diarrhea, functional digestive involvement, inflammatory bowel disease, cardiovascular diseases, allergic reactions, and cancer. Probiotic activity has been associated with *Bifidobacteria*, *Saccharomyces boulardii* and lactic acid bacteria, such as *Limosilactobacillus* (previous name: *Lactobacillus*) spp., *Lactococcus* spp., *Carnobacterium* spp., *Enterococcus* spp., *Streptococcus* spp., *Pediococcus* spp., and *Propionibacterium* spp. Probiotics can produce secondary metabolites, such as short-chain fatty acids, exopolysaccharides, organic acids, hydrogen peroxide (H_2_O_2_), and bacteriocins, which is a key mechanism through which these beneficial bacteria exert their health effects, including enhancing gut health, supporting the immune system. Moreover, probiotics can inhibit various pathogens, such as *H. pylori*^7^, *Clostridioides difficile*^8^, *Vibrio parahaemolyticus*^9^, *Escherichia coli* O157: H7^10^, and *Staphylococcus aureus*^11^.

We previously isolated *Limosilactobacillus fermentum* strain T0701 from fermented palm sap in the Songkhla Province of Southern Thailand. *L. fermentum* T0701 showed high inhibitory activity against the growth of *Acinetobacter baumannii*, *E. coli, Listeria monocytogenes, Salmonella* *typhi*, *S. enteritidis, S. flexneri*, *S. aureus,* and *E. faecalis,* and met the criteria for a potential probiotic^12,13^. However, information on its antibacterial activity against *H. pylori* is lacking*.* Therefore, the present study aimed to evaluate the potential of lyophilized cell-free supernatants (LCFS) of *L. fermentum* T0701 against *H. pylori.*

## Results

### Determination of minimum inhibitory concentration (MIC) and minimum bacterial concentration (MBC) of LCFS

*L. fermentum* T0701 LCFS showed varied antimicrobial activities against *H. pylori* ATCC43504 and five clinical *H. pylori* isolates (Table 1). The inhibition zones were 0.00 ± 0.00 and 10.67 ± 0.58 mm. The MIC and MBC of *L. fermentum* T0701 LCFS against *H. pylori* were 6.25–25.00 mg/mL and 6.25 mg/mL to > 50.00 mg/mL, respectively. This suggests that *L. fermentum* T0701 LCFS protects against *H. pylori.*

**Table 1:** Antimicrobial activity of LCFS of *L. fermentum* T0701 against *H. pylori.*

Pathogen	Agar well-diffusion assay (mm.)	MIC* (mg/mL)	MBC* (mg/mL)
*H. pylori* ATCC43504	10.67 ± 0.58	6.25	> 50
clinical *H. pylori* 006	7.00 ± 0.00	12.5	> 50
Clinical *H. pylori* 029	7.00 ± 0.00	6.25	12.5
Clinical *H. pylori* 031	7.40 ± 0.10	6.25	> 50
Clinical *H. pylori* 041	ND	3.13	6.25
Clinical *H. pylori* 117	7.00 ± 0.00	25	> 50

*Determination of minimum inhibitory concentration (MIC) and minimal bactericidal concentration (MBC).

### Scanning electron microscopy (SEM)

**Figure 1:**
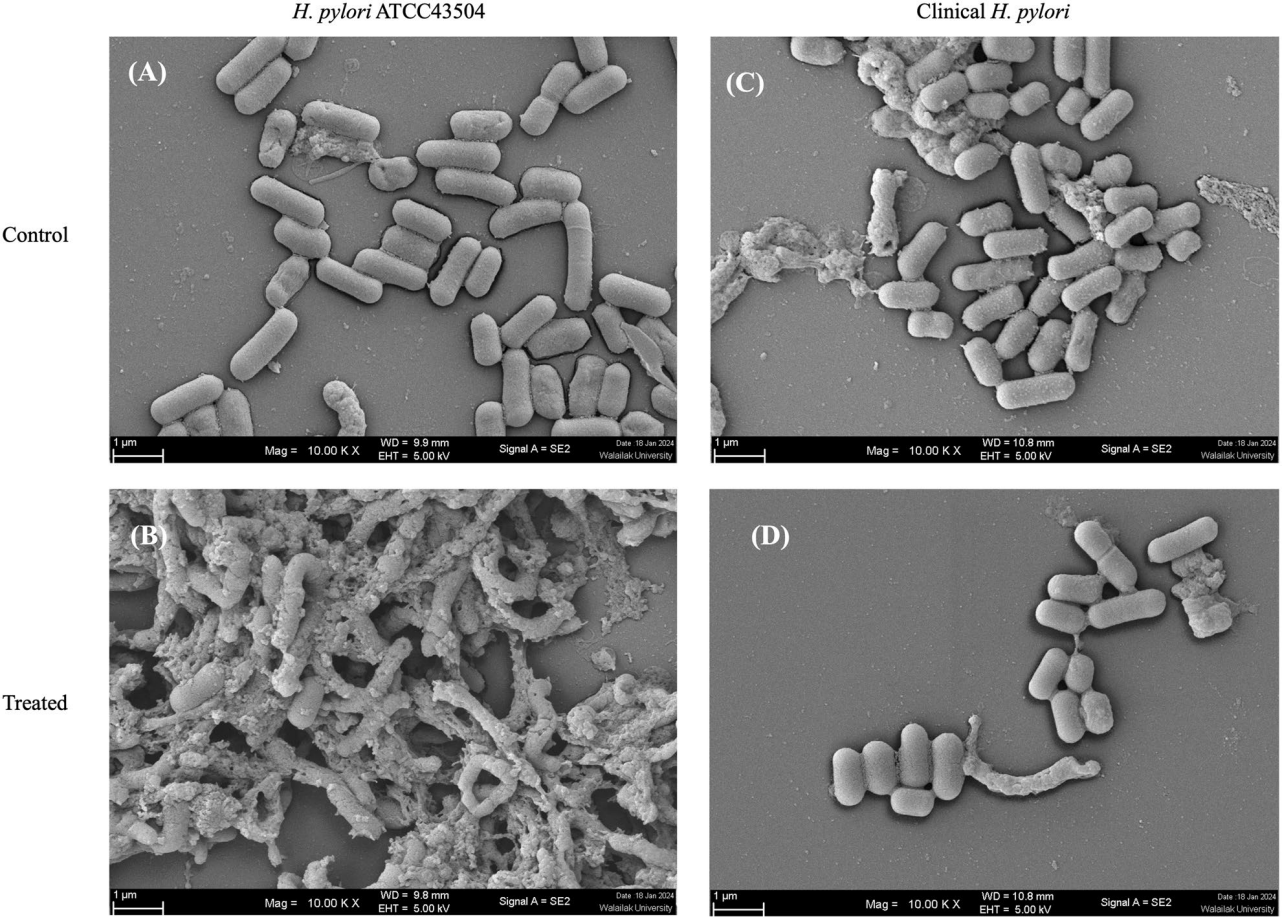
Morphology of *H. pylori* ATCC43504 (**A**,**B**) and clinical *H. pylori* (**C**,**D**) after treatment with LCFS of *L. fermentum* T0701 observed by SEM. The bacterial cells were treated with the LCFS at MIC. MRS was used as a negative control. Magnifications were revealed 10,000×. *LCFS* lyophilized cell-free supernatant, *MIC* minimum inhibitory concentration, *MRS* de Man, Rogosa and Sharpe.

The effect of the LCFS on the morphology of *H. pylori* was observed using SEM. The result showed that the normal bacterial cells (regular shapes and homogenous cell surface) appeared in the control groups of the *H. pylori* (Fig. 1A) and clinical *H. pylori* isolates (Fig. 1C). Notably, both *H. pylori* ATCC43504 and clinical *H. pylori* isolates treated with 1 × MIC of *L. fermentum* T0701 LCFS showed damaged cells compared with that in the control (Fig. 1B,D). The bacterial cells exhibited a rough, pitted, and irregular appearance along with a modified cell structure. Additionally, certain cells appeared damaged and displayed missing or ruptured membranes. Our findings suggest that the LCFS of *L. fermentum* T0701 interferes with the disruption of *H. pylori*, leading to the release of intracellular contents.

### Cell viability assay

The results of the in vitro toxicity evaluation of AGS cells using the 3-(4,5-Dimethylthiazol-2-yl)-2,5-diphenyltetrazolium bromide (MTT) assay are shown in Supplementary Table 1. The results indicated that 0.625–10 mg/mL of *L. fermentum* T0701 LCFS exhibited nontoxic effects against AGS cells. The cell viability was 91.52 ± 1.10% to 118.58 ± 12.10%. Thus, the LCFS was considered safe.

### Anti-adhesion of *H. pylori* to AGS cells by LCFS

We investigated whether *L. fermentum* T0701 LCFS could inhibit the adhesion of *H. pylori* to AGS cells. The results showed that the *L. fermentum* T0701 LCFS significantly reduced *H. pylori* adhesion to AGS cells by 74.23 ± 0.60% when compared with that with *H. pylori* infection alone (Supplementary Fig. 1). Moreover, de Man, Rogosa and Sharpe (MRS) medium did not inhibit *H. pylori* adhesion. These results suggest that *L. fermentum* T0701 LCFS adheres *H. pylori* to AGS cells.

### Effect of co-culture of LCFS and *H. pylori* on AGS cells

**Figure 2:**
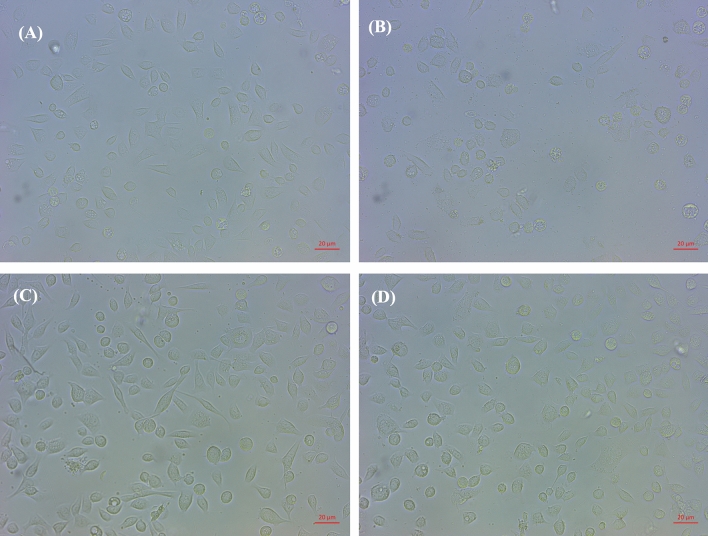
Comparison of cytotoxic effects of individual *H. pylori* ATCC43504, LCFS of *L. fermentum* T0701, and co-culture of the LCFS and *H. pylori* on AGS cells. (**A**) AGS cells, (**B**) AGS cells treated with *H. pylori* ATCC43504, (**C**) AGS cells treated with LCFS of *L. fermentum* T0701, (**D**) AGS cells treated with co-culture of LCFS and *H. pylori*. *LCFS* lyophilized cell-free supernatant.

**Figure 3:**
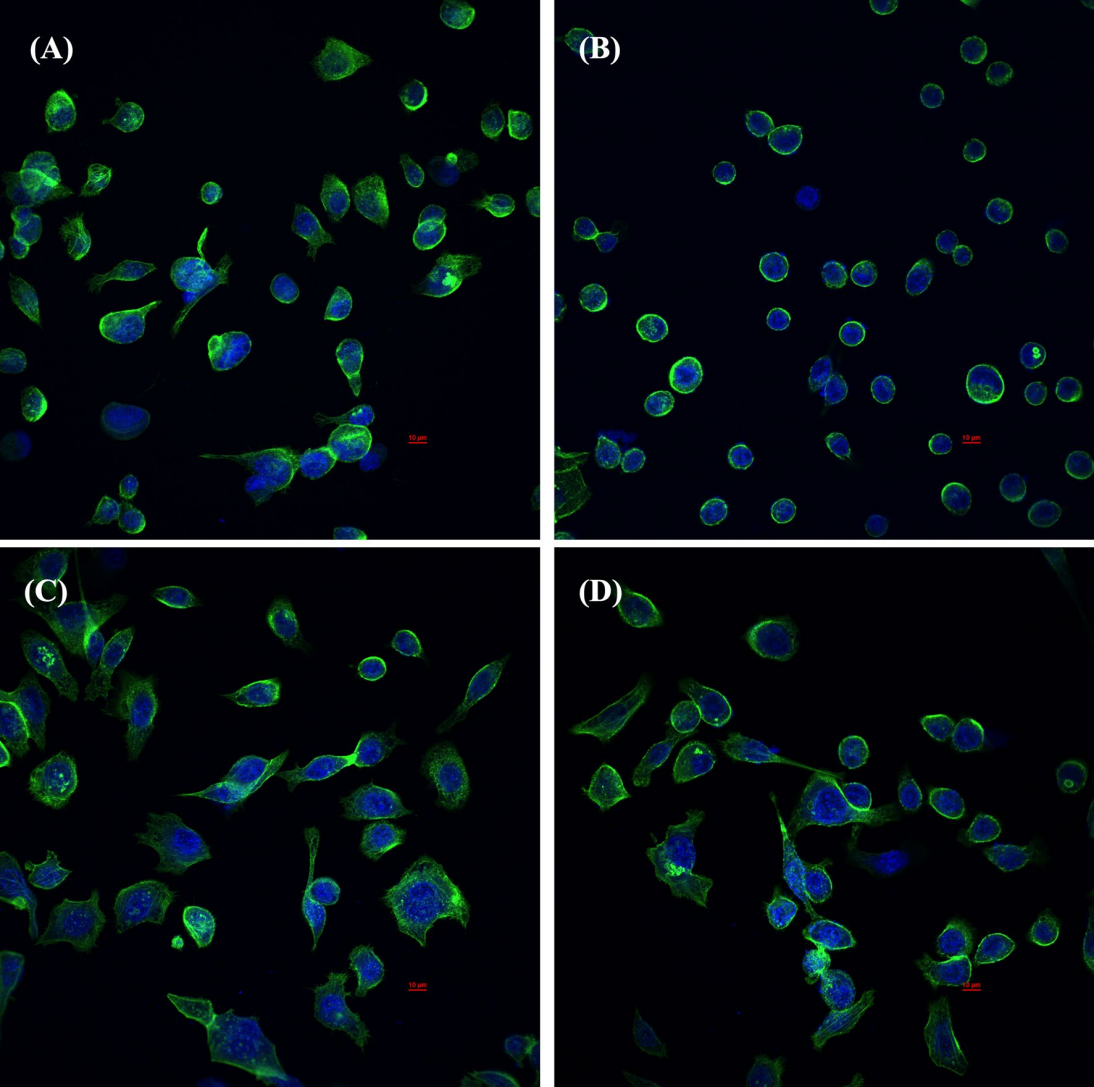
Immunofluorescence images obtained by confocal scanning laser microscopy of AGS cells after 24 h with individual *H. pylori* ATCC43504, LCFS of *L. fermentum* T0701, and co-culture of the LCFS and *H. pylori* on AGS cells. (**A**) AGS cells, (**B**) AGS cells treated with *H. pylori* ATCC43504, (**C**) AGS cells treated with LCFS of *L. fermentum* T0701, (**D**) AGS cells treated with co-culture of LCFS and *H. pylori.* DAPI-stained nucleus (blue, excited at 405 nm). F-actin stained with Phalloidin-Alexa-Fluor-488 probe (green, excited at 490 nm). *LCFS* lyophilized cell-free supernatant.

To compare the effect of *L. fermentum* T0701 LCFS on *H. pylori* infection in AGS cells, AGS cells were co-infected with *H. pylori* and LCFS for 24 h. After infection, AGS cells were examined under inverted and holotomographic microscopes. The results showed that the morphology of AGS cells was relatively normal in *L. fermentum* T0701 LCFS-treated cells (Fig. 2C) compared with that of the negative control (Fig. 2A). AGS cells were round and became spherical when treated with *H. pylori* ATCC43504 (Fig. 2B). Notably, AGS cells treated with a co-culture of LCFS and *H. pylori* remained stable and cell rounding decreased (Fig. 2D) when compared with AGS cells treated with *H. pylori*. The morphology of AGS cells was observed under a holotomographic microscope. We found that the treatment of AGS cells with *H. pylori* led to changes in the morphology of the cells when compared with normal cells. The cells formed debris, which resulted in cell death. AGS cells co-cultured with LCFS and *H. pylori* were slightly damaged. However, it was more normal than that in AGS cells treated with *H. pylori*. Furthermore, *F*-actin detection was detected using immunofluorescence. The results showed that AGS cells in the control group exhibited a typical *F*-actin cytoskeleton and an imbibed nucleus similar to those of normal cells (Fig. 3A). AGS cells treated with *H. pylori* showed a loss of *F*-actin cytoskeleton-mediated interconnections between cells and condensed nuclei, indicating the initial stage of apoptosis. The cells became rounded and tight junctions were disrupted (Fig. 3B). The images of AGS cells with *L. fermentum* T0701 LCFS were similar to those of the control group, and *F-*actin showed a normal morphology (Fig. 3C). Moreover, The AGS cells treated with co-culture of LCFS and *H. pylori* were less damaged like normal morphology when compared with AGS cells that were treated with *H. pylori* (Fig. 3D). Overall, these results suggested that *L. fermentum* T0701 LCFS reduced the effects of *H. pylori* by inhibition.

### In silico safety evaluation and bacteriocin identification

**Figure 4:**
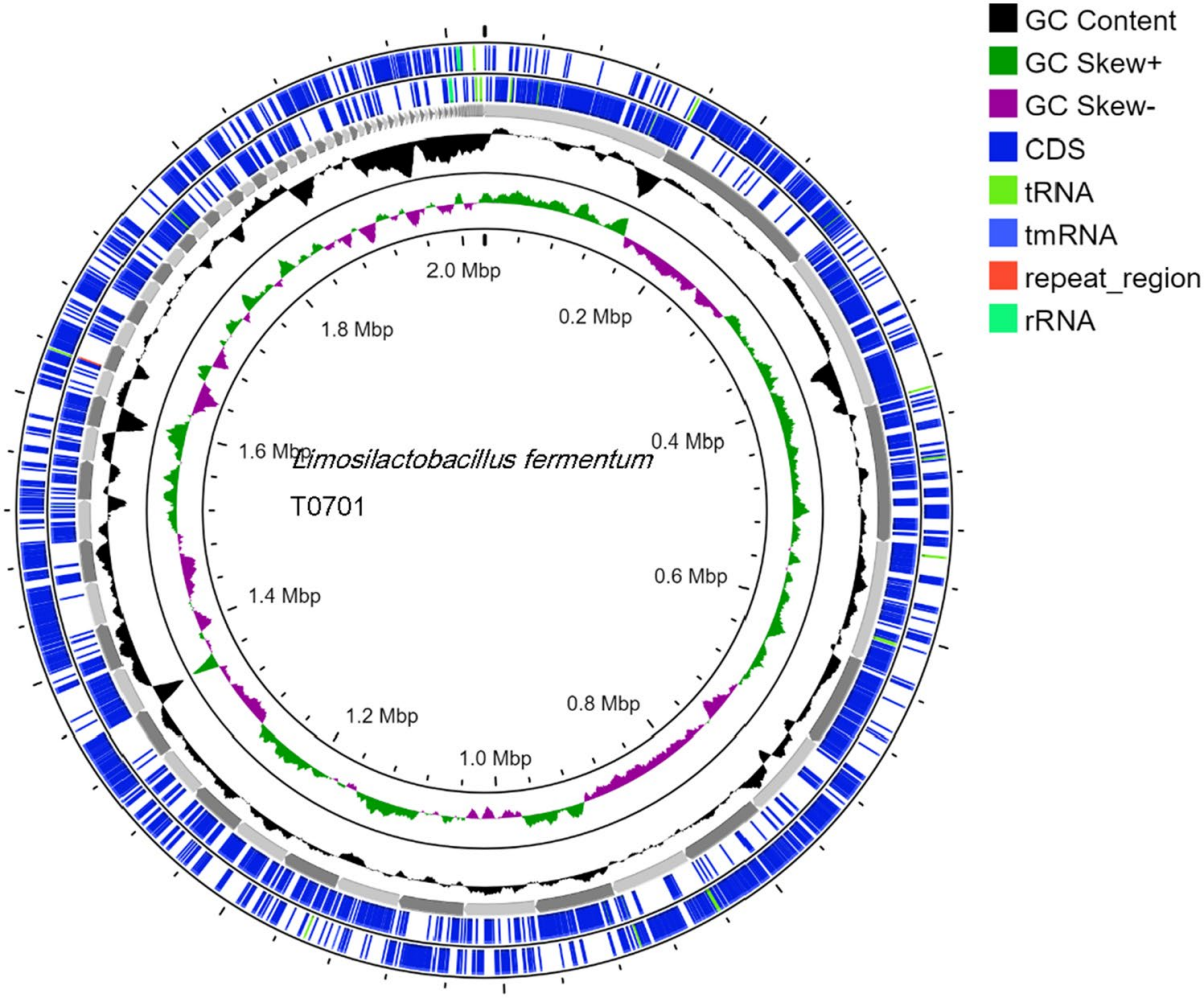
Circular genome map of *Limosilactobacillus fermentum T0701.*

In our analysis, we utilized the ABRICATE tool, a widely used bioinformatics software, to detect virulence genes by searching the VFDB (Virulence Factor Database). We applied stringent criteria with a Minimum DNA %identity of 80 and Minimum DNA %coverage of 80 during the examination. Despite these parameters, we did not identify any virulence genes in the analyzed samples. Furthermore, analysis of various biological features of this strain revealed predominant functions related to carbohydrates, amino acids, nucleotides, cofactors, and stress responses. Although these genes are commonly acknowledged as virulence factors in pathogens that support their survival in the host environment under physiological stress and facilitate adaptation to diverse conditions, the lack of such genes in T0701 addresses safety concerns. The absence of genes associated with virulence factors in strain T0701 suggested no harm to humans, confirming the lack of virulence genes (Table 2 and Fig. 4).

**Table 2:** Genome statistics of *L. fermentum* T0701.

Features	Size
Genome size (bp.)	2,026,203
Contigs	121
GC content (%)	51.86%
Number of CDS	1967
tRNA	48
rRNA	4
tmRNA	1
Repeat region	1
Bacteriocin-liked encoding gene	0

No antimicrobial resistance genes were identified in the ResFinder database, which aligned with previous safety assurances. Antibiotic susceptibility testing confirmed T0701 susceptibility to a range of antibiotics, including chloramphenicol, kanamycin, and penicillin. Additionally, examination of the T0701 strain using the MGEFinder database revealed four insertion sequences without encoded genes, indicating the potential absence of transmission capability (Table 2 and Fig. 4). *L. fermentum* T0701 contains one intact and one incomplete phage. It contained two prophage regions (Supplementary Table 2).

We manually performed a BLASTP search against the BAGEL4 database to identify bacteriocins within the genome. Our investigation showed that the three proteins encoded by the T0701 strain displayed similarity to bacteriocin-like proteins, with a coverage of 100% and similarity of over 79%, as outlined in Supplementary Table 3. Their potential as targeted antimicrobial agents, particularly in inhibiting the *H. pylori* pathogen.

## Discussion

*H. pylori* is the most common chronic bacterial infection worldwide and the greatest risk factor for gastric cancer. It remains the world's leading cause of cancer-related deaths. Moreover, it can cause chronic gastritis, peptic ulcers, and mucosa-associated lymphoid tissue lymphomas. In recent years, the use of PPIs in combination with antibiotics has significantly improved *H. pylori* treatment. However, there are substantial challenges in managing *H. pylori* infections owing to the increasing incidence of antibiotic resistance and the emergence of drug-resistant strains^14^. Probiotics and postbiotics are being explored as alternative therapeutic options for the treatment and prevention of *H. pylori* infections. Therefore, this study focused on evaluating the antibacterial potential of the LCFS of *L. fermentum* T0701 against *H. pylori*.

Probiotics are dietary supplements that consist of live, nonpathogenic microorganisms when administered in adequate quantities and are known to enhance the health status of the host^6^. Moreover, a postbiotics are defined as, “any factor resulting from the metabolic activity of a probiotic or any released molecule capable of conferring beneficial effects to the host in a direct or indirect way,” with various therapeutic applications, especially antimicrobial effects, by using different mechanisms^15^. In our previous report^12^, the LCFS of *L. fermentum* T0701 isolated from fermented palm sap exhibited antimicrobial, antibiofilm, biofilm eradication, antioxidant, and anti-inflammatory activities by reducing nitric oxide production. In the present study, The *L. fermentum* T0701 LCFS strongly inhibited *H. pylori*, as revealed by agar well assays, followed by MIC and MBC assays. Various probiotics exert inhibitory effects on *H. pylori* through distinct mechanisms^16^, including *L. johnsonii* No. 1088^17^, *L. pentosus* SLC13, and *L. gasseri* BCRC14619T^18^. Other reports have shown that *L. rhamnosus* GMNL-74, L. *L. acidophilus* and GMNL-185 can inhibit *H. pylori* infection and exhibit anti-inflammatory activities^19^. *L. fermentum* strains are known to have beneficial effects on human health^20^. They synthesize a range of secondary metabolites with antimicrobial properties, including bacteriocins, organic acids, ethyl alcohol, short-chain fatty acids, and hydrogen peroxide^21^. These peptides, which are generated by ribosomes, can disrupt the cell membrane or cause breakdown of the cell wall. However, the specific mechanism of action of some of these peptides remains unclear^20^. The *L. fermentum* T0701 LCFS destroyed the cell wall of *H. pylori* as observed using SEM.

In this study, we found that *L. fermentum* T0701 carries the lantibiotic immunity ABC transporter MutE/EpiE family permease subunit, lantibiotic protection ABC transporter ATP-binding protein, and the nisin biosynthesis regulatory protein NisR. The lantibiotic immunity ABC transporter MutE/EpiE family permease subunit is not directly involved in antimicrobial activity, but plays a crucial role in providing immunity to the host organism against the lantibiotic it produces. l-Antibiotics are a class of peptide antibiotics produced by certain bacteria that are highly effective against a broad range of bacterial pathogens. These antibiotics function by binding to the cell walls of target bacteria, disrupting cell wall synthesis or forming pores in the bacterial membrane, leading to cell death^22,23^. The lantibiotic protection ABC transporter ATP-binding protein is a key component of the self-protection mechanism of lantibiotic-producing bacteria, enabling them to safely produce lantibiotics that inhibit pathogens without the protein itself directly engaging in pathogen inhibition^24^. Although NisR itself does not inhibit pathogens, its regulation of nisin production is critical for the antimicrobial defense strategy of *L. lactis*. By controlling nisin production, NisR indirectly contributes to the inhibition of pathogenic bacteria in the environment where *L. lactis* resides. Once produced and secreted, nisin can bind to the cell wall precursors of susceptible bacteria, form pores in their membranes, and initiate cell death, thereby inhibiting the growth of pathogens^25^.

Furthermore, *L. fermentum* is acknowledged as be safe and listed in the official registers of European, American, and Chinese food safety authorities^26–28^. Prophages are commonly found in probiotic strains utilized in dairy fermentation, including *Lactococcus, Bifidobacterium*, and *Limosilactobacillus*^29^. Prophages are frequently detected in *Limosilactobacillus* strains, with a typical range of one to five prophages per genome^30,31^. This suggests that prophages are prevalent in the genomes of probiotic bacteria and emphasizes their significance in the context of dairy fermentation and related applications. In addition, *L. fermentum* T0701 did not carry antimicrobial resistance or virulence genes, suggesting that it is safe.

The adhesion of *H. pylori* to epithelial cells is a critical step in its colonization and infection of the human stomach. Most of these connections are facilitated by outer membrane proteins (OMPs) that function as adhesins. The genome of *H. pylori* has approximately 30 genes encoding OMPs^2,16^. The present study showed that *L. fermentum* T0701 LCFS reduced the adhesion of *H. pylori* to AGS cells. Similarly, *Limosilactobacillus* and *S. boulardii,* can prevent *H. pylori* from adhering to gastric epithelial cells by competing for binding sites, such as asialo-GM1 and sulfatide receptors^19^. Other probiotics such as *L. acidophilus, L. johnsonii,* and *L. salivarius* also exhibit similar properties, preventing *H. pylori* colonization through specific adhesion molecules^19^. Other studies have shown that cell-free supernatants of *L. pentosus* SLC13, *L. rhamnosus* LGG, and *L. gasseri* BCRC 14619 T can reduce *H. pylori* adhesion to GES-1 cells by 54.2%, 52.4%, and 38.0%, respectively^18^. Thus, the anti-adhesion effect is crucial for inhibiting *H. pylori* colonization and stomach infections, providing a potential complementary approach to traditional *H. pylori* eradication therapies.

Upon entry into cells, CagA can alter cell signaling pathways, promote inflammation, and potentially contribute to carcinogenesis. VacA causes cellular damage by forming pores in the cell membrane, leading to cell death and contributing to the pathology of *H. pylori*-associated diseases^2,5,32^. In the previous study, *L. rhamnosus* JB3 had multiple mechanisms to disrupt *H. pylori* pathogenesis and the cellular responses of AGS cells caused by infection, to combat the infection^33^. *L. reuteri 2892* exhibited protective properties against *H. pylori*-induced gastritis in animal models by effectively suppressing the expression of virulence factors in the gastric mucosa^34^. Another study demonstrated that *L. fermentum* inhibits *H. pylori* in AGS cells through several key actions; *L. fermentum* UCO-979C has been shown to beneficially modulates the innate immune response triggered by *H. pylori* infection and reduce the adhesion of *H. pylori* to gastric epithelial cells. It achieves this by substantially reducing the production of inflammatory cytokines and chemokines while increasing the levels of immunoregulatory cytokines and reducing cell damage^35^. Moreover, *L. fermentum* MN-LF23 reduces *H. pylori* infection in infected mice and ameliorates *H. pylori*-induced gastric mucosal damage and lymphocyte infiltration^36^. Similarly, the results of our study showed that *L. fermentum* T0701 LCFS may secrete antibacterial agents that reduce the cytotoxic effects of *H. pylori* protection of AGS cells.

To the best of our knowledge, *L. fermentum* T0701 LCFS exhibited significant anti-*H. pylori* activity, inhibition of bacterial adhesion to AGS cells, and bactericidal properties. Moreover, it is safe and non-toxic to AGS cells, indicating its potential as a therapeutic agent against *H. pylori* infection. These findings suggest that LCFS could be an effective non-antibiotic strategy for managing *H. pylori* infections, potentially reducing the burden of antibiotic resistance and offering a complementary approach to current treatment regimens. Further research is warranted to identify the specific molecules responsible for anti-*H. pylori* activity and to explore the clinical potential and mechanisms underlying *L. fermentum* T0701 LCFS effects against *H. pylori*.

## Methods

### Bacterial strains and culture conditions

*L. fermentum* T0701 was isolated and characterized as a potential probiotic in a previous study^13^. Briefly, the isolate was grown in MRS broth (HiMedia, Mumbai, India) at 37 °C for 24 h. After that, all isolates were stored at − 80 °C in 30% (v/v) glycerol (Sigma, Steinheim, Germany) until further use.

*H. pylori* ATCC43504 strain was purchased from the American Type Culture Collection and 10 clinical *H. pylori* isolates were collected from Songklanagarind hospital. These strains were cultured on blood agar (HiMedia) containing 7% horse blood, and the agar plates were incubated at 37 °C for 48 h under microaerophilic conditions. The colonies were transferred to brain heart infusion broth (HiMedia) and incubated at 37 °C for 18 h. Each strain was stored at − 80 °C in brain heart infusion broth with 30% glycerol until further use.

### Preparation of cell-free supernatants (CFS) and lyophilization

CFSs were prepared according to the method described by Sornsenee et al.^12^. *L. fermentum* T0701 was cultured in 250 mL of MRS broth and incubated at 37 °C for 24 h under anaerobic conditions. The bacterial cultures were then centrifuged at 10,000×*g*. The supernatant was sterile filtered using a 0.2 µm filter (Sigma, Steinheim, Germany). CFS of *L. fermentum* T0701 and only MRS medium as MRS control were frozen at − 80 °C for 24 h. The samples were lyophilized (Lyophilization Systems, Inc, USA) from − 40 to − 30 °C, 0.2 mbar. The entire freeze-drying process was performed in 24 h, and the freeze-dried powders were stored at − 20 °C. They were then rehydrated with sterile deionized water prior to use.

### Agar well-diffusion assay

This assay was performed in accordance with Romyasamit, et al.^8^, with minor modifications. Briefly, LCFS of *L. fermentum* T0701 were resuspended in sterile deionized water (100 mg/mL). Each *H. pylori* ATCC43504 and 5 clinical *H. pylori* isolates (1.5 × 10^8^ CFU/mL) were spread on blood agar containing 7% horse blood and were cut out of the agar using sterile Pasteur pipettes. Then 50 μL of LCFS were added into the agar wells. The plates were incubated at 37 °C for 72 h under microaerophilic conditions and were inspected for the presence of inhibition zones. The antibacterial activity was expressed as the mean of inhibition diameters (mm) produced by LCFS. The tests were performed in triplicate.

### Determination of MIC and MBC

The antimicrobial efficacy of LCFS against *H. pylori* ATCC43504 was evaluated employing the microdilution method in 96-well plates, following the Clinical and Laboratory Standards Institute 2022 guidelines^37^. 100 µL of serial dilution of LCFS (100–3.125 mg/mL) were prepared in Mueller Hinton broth (MHB) (HiMedia). The *H. pylori* ATCC43504 suspensions were adjusted to McFarland turbidity standard 2 and then 100 µL was inoculated into each well. The plates were incubated at 37 °C for 72 h under microaerophilic conditions. To determine the MIC, the colorimetric dye resazurin was used, which serves as an indicator of cell viability. After the incubation period, 20 µL of resazurin solution (0.01% w/v in sterile PBS was added to each well). The plates were then incubated for 4 h. The MIC was defined as the lowest concentration of LCFS at which a change in color from blue (oxidized resazurin) to pink (reduced resazurin) was not observed, indicating inhibition of bacterial growth.

The MBC was determined for extracts with noteworthy MIC values by inoculating the culture onto Brucella agar containing 7% horse blood plates. After incubation in microaerophilic conditions at 37 °C for 72 h, the colonies formed were subsequently computed. The MBC was defined as the lowest concentrations of the LCFS, inducing complete inhibition of colony formation of the test bacteria at the latter cultivation.

### SEM

The effects of LCFS of *L. fermentum* T0701 inhibit *H. pylori* ATCC43504 on morphology of *H. pylori* cells were determined using SEM according to Kim et al.^38^ Briefly, *H. pylori* ATCC 43504 was cultivated MHB at 37 °C for 48 h. The bacterial suspensions were adjusted to 2 McFarland in a centrifuge tube containing 4X MIC of LCFS, incubated at 37 °C for 24 h under microaerophilic conditions. MRS broth was used as a negative control. After that the samples were then centrifuged at 10,000 rpm for 5 min and washes with phosphate-buffered saline (PBS) and centrifuged. Both control and LCFS-treated cells were dropped on a sterile glass coverslip (0.5 cm × 0.5 cm), and air dried. All samples were fixed using 2.5% (v/v) glutaraldehyde (Sigma-Aldrich, St Louis, MO, USA) in 0.1 M phosphate buffer for 24 h at 4 °C. Then, the cells were dehydrated in a graded ethanol series (40%, 60%, 80%, and 95% v/v) for 15 min each session, followed by two dehydration steps in 100% ethanol (Thermo Fisher Scientific, NY, USA) for 15 min followed by gold coating. Morphology of the bacteria after treatment with the LCFS was observed by field emission scanning electron microscope (Oxford Instruments, Quanta, Japan).

### Cell culture

AGS cells (Passage number 5), a human gastric adenocarcinoma epithelial cells, ATCC CR-1739 (were purchased from American type culture collection, USA. AGS cells were cultured in Ham’s F12 (Gibco, Thermo Fisher Scientific) with 10% fetal bovine serum (Gibco) and 1% penicillin–streptomycin solution (Gibco) at 37 °C in 5% CO_2_. The AGS cells were subcultured and plated at 90% confluency.

### Cell viability assay

The MTT assays were performed to assess the effect of *L. fermentum* T0701 LCFS on the viability of AGS cells according to a previously established procedure^12^, with slightly modifications. Briefly, AGS cells were seeded onto 96-well microplates at a density of 5 × 10^4^ cells/mL and incubated at 37 °C in a 5% CO_2_ incubator for the cytotoxicity assays. The cells were then treated with LCFS (10.00–0.625 mg/mL) and incubated at 37 °C for 24 h. After incubation, the supernatants were discarded and the cells were washed with PBS. A volume of 100 µL of MTT solution (Sigma-Aldrich; 0.5 mg/mL in PBS) was added to each well and incubated for 4 h in the dark after removing the treatment mixture from each well. The formazan crystals were dissolved by adding 200 µL of dimethylsulfoxide solution (Sigma-Aldrich). The optical density was measured at 570 nm using a microplate reader. The experiment was repeated three times. The percentage cell viability was calculated using the following equation:

$${\text{Cell}}\;{\text{viability}}\;(\% ) = \left( {{\text{A}}_{{{\text{test}}}} {\text{/A}}_{{{\text{control}}}} } \right) \times 100,$$

where A_test_ is the absorbance of the cells incubated with the medium containing LCFS, and A_control_ is the absorbance of the cells alone.

### Anti-adhesion of *H. pylori* to AGS cells by LCFS

This experiment was performed as described by Lim et al.^39^ with minor modifications. Briefly, AGS cells were seeded onto 24-well microplates at 1 × 10^5^ cells/mL and incubated at 37 °C in a 5% CO_2_ until a confluent monolayer formed, which typically occurred within 48 h. LCFS (5 mg/mL) was resuspended in Ham’s F12 medium supplemented with or without *H. pylori* (10^8^ CFU/mL) and then incubated for 2 h under microaerophilic conditions with stirring (200 rpm). The suspension was then added to a cell culture plate and incubated for 4 h to allow *H. pylori* to adhere to the cell lines. To assess the adhesion ability of *H. pylori*, cells were washed three times with PBS and lysed with 0.25% trypsin–EDTA for 5 min. The lysates were serially diluted in PBS and plated onto BA-containing 7% horse blood plates at 37 °C under microaerophilic conditions for 72 h. The number of *H. pylori* cells adhering to AGS cells was calculated as follows:

$$\% \;{\text{adhesion}}\;{\text{ability}} = \left( {{\text{V}}1 \times 100} \right)/{\text{V}}0,$$

where V0 is the initial viable count and V1 is the viable count adhered to the AGS cells after incubation.

### Co-culture of LCFS and *H. pylori* with AGS cells

The method was performed according to the procedure reported by Romyasamit et al.^8^ with minor modifications. The cytopathic effects of *H. pylori* on AGS cells were evaluated. First, AGS cells were seeded onto 24-well microplates at 1 × 10^5^ cells/mL and incubated at 37 °C in a 5% CO_2_ until a confluent monolayer formed. LCFS (5 mg/mL) were resuspended in Ham’s F12 medium supplemented with or without *H. pylori* (10^8^ CFU/mL) and then incubated for 2 h under microaerophilic conditions with stirring (200 rpm). The supernatant was added to each well. The plates were incubated for 24 h at 37 °C in 5% CO_2_. The cells were examined under an inverted microscope and a commercial holotomographic microscope (HT-2H, Tomocube Inc.) for morphological changes.

### Immunofluorescence assay

AGS cells treated with or without the co-culture of LCFS and *H. pylori* were analyzed using confocal microscopy. Immunofluorescence assays were conducted as described previously^8,40^. Briefly, AGS cells were seeded in eight-well plates and incubated until they reached a confluent state. The supernatant was then removed, and each supernatant containing LCFS was added to the wells with *H. pylori* or Ham’s F12 media (negative control) and incubated for 24 h at 37 °C in 5% CO_2_. Subsequently, AGS cells were fixed with 300 μL of 3.7% formaldehyde for 10 min. The samples were washed four times and permeabilized with 0.1% Triton X-100 for 10 min. Non-specific binding sites were blocked by treatment with 1% BSA for 15 min. The Phalloidin-Alexa-Fluor-488 probe (Invitrogen, USA) was used at a dilution of 1:40, which toward F-actin was added, and incubated for 1 h at 4 °C in darkness. The nuclei of AGS cells were stained with 300 nM of DAPI (Sigma Chemical Co.) and incubated for 20 min. Finally, the samples were washed and 50 μL of anti-fade mountants (Invitrogen) was added prior to visualization under a Super-Resolution Laser Scanning Confocal Microscope; SR-LSCM (ZEISS, Germany) using a 63×/1.4 oil objective.

### DNA isolation and whole-genome sequencing

Probiotics were previously isolated from fermented palm sap^13^. A single colony of *L. fermentum* T0701 was cultivated in MRS broth (HiMedia) at 37 °C for 24 h under anaerobic conditions. Genomic DNA was purified and extracted using a DNeasy extraction kit (QIAGEN, Hilden, Germany) following the manufacturer’s instructions. The purity of the DNA was estimated using a spectrophotometer by measuring the absorbance at 260 and 280 nm (A260/A280) and 1.5% agarose gel electrophoresis. Purified genomic DNA was sequenced using a with NextSeq™ 550 System (Illumina, Inc., San Diego, CA, USA). The quality of the reads and low-quality reads were removed using Trim Galore (Galaxy Version 0.6.3) with default parameters.

### Genome assembly and annotation

A dataset of 150-bp paired-end reads totaling 1 Gb pair was obtained from the sequence provider. Subsequently, the sequence reads were assembled and annotated using the comprehensive BacSeq v1.0 pipeline^41^ for assembly^42^, annotation^43^, and assessment of the quality and completeness of the genome assembly^44,45^. An expected BUSCO completeness score exceeding 95% was targeted as a measure of assembly completeness. Mobile genetic elements, prophages, and antimicrobial resistance genes were identified using mobileOG-db^46^, Phaster^47^, VirulenceFinder^48^, and ResFinder web-based tools^49^. The detection of genes encoding bacteriocins involved a sequence similarity search, with the results presented using the BAGEL4 webserver^50^.

### Statistical analysis

All data from this study are expressed as the mean ± standard deviation, and a minimum of three independent experiments were performed. Statistical significance was assessed between two groups using one-way ANOVA and *t*-tests. Values of *p* < 0.05 were considered significant differences.

### Supplementary Information

Supplementary Information.

## Data Availability

Data included in article/supplementary material/referenced in article.
