# Supplementary material for: Lyophilized cell-free supernatants of Limosilactobacillus fermentum T0701 exhibited antibacterial activity against Helicobacter pylori
Source: Sci Rep. 2024 Jun 13;14:13632. doi: 10.1038/s41598-024-64443-4 (PMC11176309; doi:10.1038/s41598-024-64443-4)
Supplement: Supplementary file 1 — Supplementary Information. [file 41598_2024_64443_MOESM1_ESM.docx]

**Supplementary Table 1.** Cytotoxicity of the LCFS of *L. fermentum* T0701 in AGS cells

| Concentation of LCFS (mg/mL) | Cell viability (%) |
| --- | --- |
| 10 | 91.52 ± 1.10 |
| 5 | 102.41 ± 9.24 |
| 2.5 | 102.92 ± 10.42 |
| 1.25 | 103.09 ± 7.85 |
| 0.625 | 118.58 ± 12.10 |

**Supplementary Table 2.** Predicted prophage regions in the genome of *L paracasei* T0701.

| **Contig** | **Completeness** | **Score** | **Total proteins** | **Region Position** | **Most common phage** | **GC (%)** |
| --- | --- | --- | --- | --- | --- | --- |
| NODE_2_length_137747_cov_247.656723 | incomplete | 10 | 10 | 79243-87714 info_outline | PHAGE_Nodula_vB_NspS_kac65v151_NC_048756(2) | 0.5123 |
| NODE_3_length_132037_cov_186.274235 | intact | 150 | 47 | 90384-129768 info_outline | PHAGE_Lactob_LF1_NC_019486(16) | 44.95% |

**Supplementary Table 3.** Bacteriocin Identification in the Genome

| **Bacteriocin** | **Locus tag** | **E-value** | **Coverage** | **Identity** | **Sequences** |
| --- | --- | --- | --- | --- | --- |
| Lantibiotic immunity ABC transporter MutE/EpiE family permease subunit | AKIDFCNF_01973 | 5.02E-47 | 100% | 86.1% | MKQRLGLGMAILTEPEFLILDEPTNGLDPDGINELLDLMRKLKESGTTILVSSHQLHEVSKVADKIVILNQGQIRYDNLNNDDSDLEQTFFRIVHGG |
| Lantibiotic protection ABC transporter ATP-binding protein | AKIDFCNF_01974 | 4.62E-47 | 100% | 76.6% | QVLVASILFVTFLKLVSLLYVGIIEVSAVKVLLTLCLMLVAVSWNLPLLYLLSNWINPYILLIGNTFICLLVAPLIAQTPFWFLFPFTYHYK |
| Nisin biosynthesis regulatory protein NisR | AKIDFCNF_01976 | 1.12E-39 | 100% | 73.6% | MKQRLGLGMAILTEPEFLILDEPTNGLDPDGINELLDLMRKLKESGTTILVSSHQLHEVSKVADKIVILNQGQIRYDNLNNDDSDLEQTFFRIVHGG |


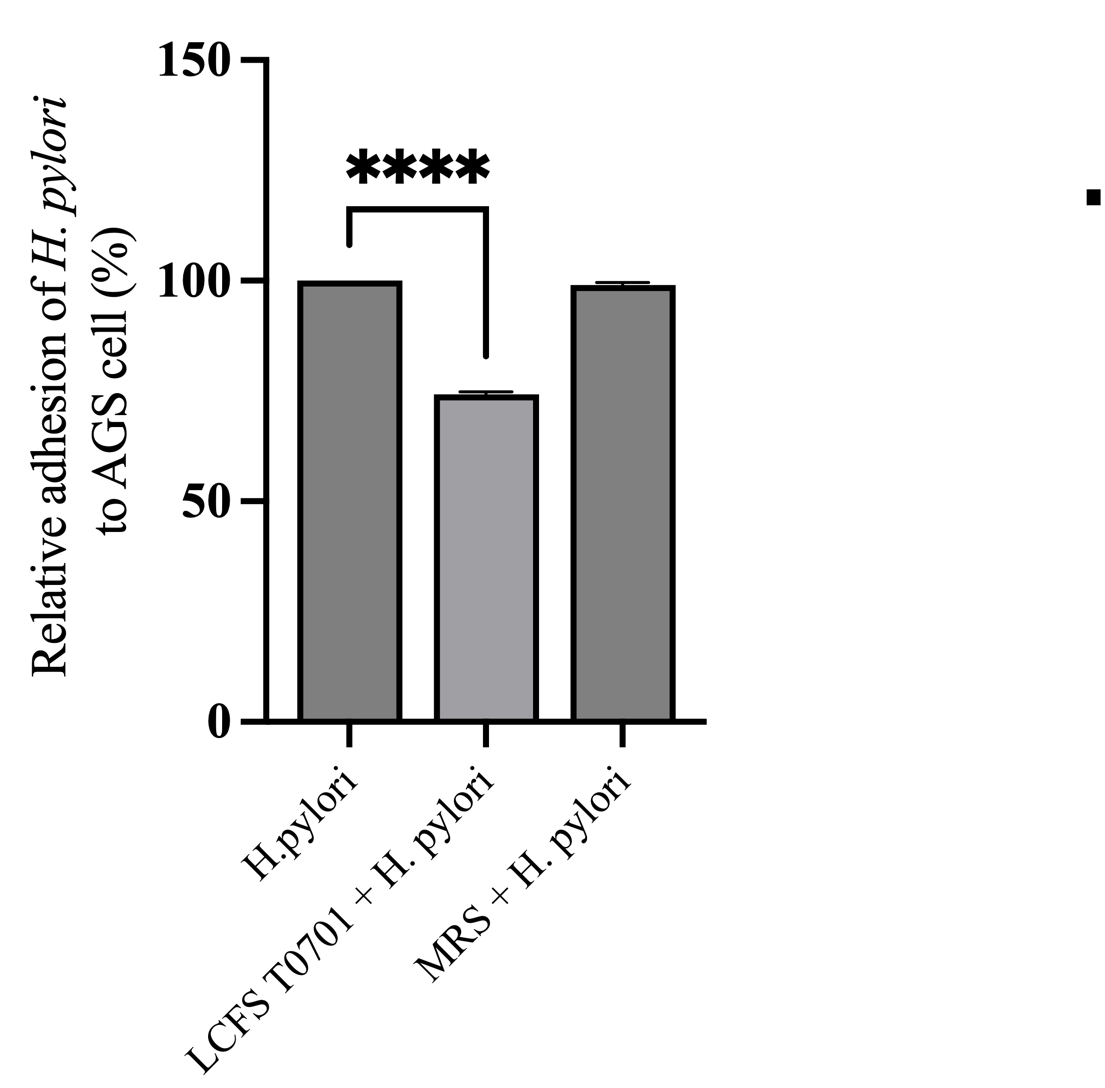


**Supplementary figure 1.** Adhesion effects of LCFS of *L. fermentum* T0701 **on** *H. pylori.* AGS cells were treated with co-culture of LCFS of *L. fermentum* T0701 and *H. pylori* ATCC43504 for 4 h. * *p* < 0.05.
